# Variables Associated with Endometriosis-related Pain: A Pilot Study using a Visual Analogue Scale

**DOI:** 10.1055/s-0039-1679879

**Published:** 2019-03

**Authors:** Mauro Cozzolino, Maria Elisabetta Coccia, Giacomo Lazzeri, Francesca Basile, Gianmarco Troiano

**Affiliations:** 1 Department of Biomedical, Experimental and Clinical Sciences – Division of Obstetrics and Gynecology, University of Florence, Careggi University Hospital, Florence, Italy; 2 Universidad Rey Juan Carlos, Madrid, Spain; 3 Department of Molecular and Developmental Medicine, University of Siena, Siena, Italy; 4 Department of Human Pathology and Evolutionary Age, University of Messina, Messina, Italy; 5 University of Siena, Siena, Italy

**Keywords:** chronic pelvic pain, constipation, dyspareunia, dysmenorrhea, endometriosis, visual analogue scale, dor pélvica crônica, constipação, dispareunia, dismenorreia, endometriose, escala visual analógica

## Abstract

**Objective** Endometriosis is a complex disease, and pain is an important component of the syndrome. One of the most used methods to assess pain is the visual analogue scale (VAS). The aim of the present research was to study the pain experienced by patients who referred to our unit for endometriosis, using the VAS to understand the variables that could influence it.

**Methods** We have conducted a prospective study from February 2012 to December 2016, enrolling 388 patients who referred to a university hospital, in Florence, Italy. We have included in the present study patients during their follow-up for endometriosis; we have also included patients who underwent surgery with a histological diagnosis of endometriosis. We have collected sociodemographic and clinical information regarding age, body mass index (BMI), smoking habit, number of pregnancies, and endometriosis staging. Finally, we have administered the VAS for several symptoms.

**Results** Dysmenorrhea was the symptom associated with the highest perception of pain (mean VAS score of 5.76). The logistic regression showed that the stage of endometriosis could influence the pain associated to constipation and to dysuria. The linear regression showed that age could influence the pain associated to constipation, to dyspareunia, and to dysmenorrhea. A positive correlation was found between dysmenorrhea and chronic pelvic pain (CPP), between dysmenorrhea and dyspareunia, and between constipation and dysuria.

**Conclusion** Using a validated method, the VAS, we have studied the pain experienced by a group of patients with a history of endometriosis and observed that smoking habit and BMI did not influence the VAS scores, and that dysmenorrhea was associated with the highest perception of pain.

## Abstract (pt)

**Resumo:**

**Objetivo** A endometriose é uma doença complexa, e a dor é um componente importante da enfermidade. Um dos métodos mais utilizados para avaliar a dor é a escala visual analógica (EVA). O objetivo da presente pesquisa foi estudar a dor sentida pelas pacientes que se referiram à nossa unidade para endometriose, usando a EVA para entender as variáveis que poderiam influenciá-la.

**Métodos** Realizamos um estudo transversal de fevereiro de 2012 a dezembro de 2016, envolvendo 388 pacientes que se referiram a um hospital universitário, em Florença, Itália. Incluímos nossos pacientes do estudo durante o acompanhamento da endometriose; incluímos também pacientes que se submeteram à cirurgia com diagnóstico histológico de endometriose. Coletamos informações sociodemográficas e clínicas sobre idade, índice de massa corporal (IMC), hábito de fumar, número de gravidezes e estágio da endometriose. Finalmente, administramos a EVA para vários sintomas.

**Resultados** A dismenorreia foi o sintoma associado à maior percepção de dor (média do escore EVA de 5,76). A regressão logística mostrou que o estágio da endometriose poderia influenciar a dor associada à constipação e à disúria. A regressão linear mostrou que a idade poderia influenciar a dor associada à constipação, à dispareunia e à dismenorreia. Uma correlação positiva foi encontrada entre dismenorreia e dor pélvica crônica, entre dismenorreia e dispareunia, e entre constipação e disúria.

**Conclusão** Utilizando um método validado, a EVA, estudamos a dor sentida por um grupo de pacientes com história de endometriose e observamos que o hábito de fumar e o IMC não influenciaram os escores EVA, e que a dismenorreia foi associada à maior percepção de dor.

## Introduction

Endometriosis could be defined as the presence of endometrial-type mucosa outside the uterine cavity, and its standard diagnosis is performed through a direct visualization and histological examination of the lesions.^1^ Endometriosis has been estimated to affect up to between 10 and 15% of women of reproductive age.^2^ The prevalence of endometriosis increases dramatically to as high as between 25–50% in women with infertility, and between 30–50% of the women with endometriosis have infertility.^3^ One of the main symptoms of endometriosis is pain. Classically, there are three types of pain related to endometriosis: dysmenorrhea, deep dyspareunia, and non-menstrual chronic pelvic pain (CPP).^4^ Women with endometriosis may also experience a wide variety of other symptoms, including dyschezia, dysuria, gastrointestinal complaints, rectal bleeding with significant bowel involvement, menstrual dysfunction such as oligomenorrhea or hypermenorrhea, low back pain, and infertility.^5^ ^6^ ^7^ The pain usually begins 1 or 2 days before the expected onset of menstruation, may be unilateral or bilateral, and lasts until the end of menses. However, some women may experience a constant, debilitating pain that interferes with their daily lives.^8^

In particular, CPP causes neurological changes in the dorsal horn of the spine, resulting in neurogenic inflammation of multiple pelvic viscera, hyperalgesia, dysreflexia, a lower sensory threshold and, therefore, a greater perception of pain. Endometriosis is observed in between 71–87% of the women with CPP.^9^ ^10^ A recently published review about pain assessment in patients with endometriosis stated that the visual analog scale (VAS) was used most frequently, with a total of 167 publications identified in this search.^4^ The VAS consists of a 10-cm long horizontal line with its extremes marked as ‘no pain’ and ‘worst pain imaginable’. Each patient ticks her pain level on the line and the distance from ‘no pain’ on the extreme left to the tick mark is measured in centimeters, yielding a pain score from 0 to 10. This self-report of pain is considered as the gold standard of pain measurement.^11^

The aim of the present research was to study the pain experienced by a group of patients who referred to our unit with a history of endometriosis. Our primary objectives were: 1) to use the VAS, to measure the pain related to the following symptoms: dyschezia, dysuria, chronic pelvic pain, dyspareunia, and dysmenorrhea; 2) to use the VAS score to evaluate the impact of age, body mass index (BMI), smoking habit, and stage of the disease on pain; 3) to detect possible correlations among the symptoms in terms of VAS scores.

## Methods

### Study Design

We have conducted a prospective study from February 2012 to December 2016, enrolling patients who referred to our tertiary university hospital, in Florence.

The present study was approved and data were collected in a totally anonymous way: the patients signed an informed consent form for the clinical investigations and for the disposition of the results for scientific analysis according to privacy laws and to human rights before being enrolled.

We have included in the present study patients during their follow-up for endometriosis; we have also included patients who underwent surgery with a histological diagnosis of endometriosis. The histological diagnosis was performed by evaluating the presence of endometrial tissue outside the uterine cavity, according to the current guidelines.^12^ In the study group, the stage of endometriosis was determined using the medical records of the patients, and it was classified according to the definition of the American Society for Reproductive Medicine (ASMR).^13^ Endometriosis is classified on the basis of number, size, and superficial and/or deep location of endometrial implants, plaques, endometriomas and/or adhesions, as follows: stage I (minimal, 1–5 points), stage II (mild, 6–15 points), stage III (moderate, 16–40 points), and stage IV (severe, > 40 points).

Sociodemographic and clinical information regarding age, BMI, smoking habit, and number of pregnancies were only available when the study started; afterwards, we interviewed the patients by administering the VAS: we have used this score to describe the pain associated with dyschezia, dysuria, CPP, dyspareunia, and dysmenorrhea.

### Statistical Analysis

Data were collected in a database and processed using the software Stata 12 (StataCorp, College Station, TX, USA). The Shapiro-Wilk test was used to assess the non-normal distribution of the examined variables. Although not normally distributed, we have calculated means, medians, and standard deviations (SDs) for each variable. We have used logistic regression and the Spearman correlation test to analyze the relationship between the independent variables (stage, age, smoking habit, and BMI) and the VAS scores of each type of pain. A multiple regression was used to understand a possible correlation between the pain with the highest VAS score and the other types of pain. The level of significance was set at *p* < 0.05.

## Results

We have collected information from 388 women: our sample had a mean age of 36.65 years old (SD = 8.99; median = 37 years old; range 17–58 years old). The mean BMI was 22.5 kg/m^2^ (SD = 3.9; median = 21.55; range: 17.3–35.4). Out of the total sample, 30.67% of the women declared to be smokers, 27.84% declared to have had at least one previous pregnancy; 80.36% of the women declared that they presented with symptoms of endometriosis. In 334 women, a histological analysis was performed to classify the stage of the disease (Table 1).

**Table 1:** Stage of endometriosis

Stage	Percentage (%)
**I**	25.45
**II**	32.34
**III**	18.56
**IV**	23.65

The VAS was administered to evaluate the pain associated with the five most frequent symptoms associated to endometriosis (Table 2).

**Table 2:** Visual analogue scale scores (means, standard deviation and medians) of each symptom

Symptom	Mean score	Standard deviation	Median score
Dyschezia	1.19	2.43	0 (range 0–8)
Dysuria	0.19	1.20	0 (range 0–8)
Chronic pelvic pain	2.55	3.52	0 (range 0–10)
Dyspareunia	2.87	3.80	0 (range 0–10)
Dysmenorrhea	5.76	3.67	7 (range 0–10)

Dysmenorrhea was associated with the highest perception of pain (mean VAS score of 5.76).

### Impact of Single Independent Variables on Pain

The impact of independent variables on pain is described in Table 3.

**Table 3:** Impact of single independent variables on visual analogue scale scores of each symptom

*I – Impact of BMI*			
Symptom	Coefficient	Adjusted R^2^	*p*-value
**Dyschezia**	−0.02	0.0008	0.406
**Dysuria**	−0.008	−0.0017	0.567
**Chronic pelvic pain**	0.002	−0.0026	0.949
**Dyspareunia**	0.012	−0.0024	0.803
**Dysmenorrhea**	0.073	0.0037	0.120
***II – Impact of smoking habit***			
**Symptom**	**OR**	**Pseudo R^2^**	***p***-**value**
**Dyschezia**	0.807	0.0015	0.442
**Dysuria**	1.301	0.0017	0.679
**Chronic pelvic pain**	0.795	0.0019	0.320
**Dyspareunia**	1.384	0.0041	0.146
**Dysmenorrhea**	0.657	0.0065	0.096
***III – Impact of stage***			
**Symptom**	**OR**	**Pseudo R^2^**	***p*** **-value**
**Dyschezia**	1.641	0.0471	0.000
**Dysuria**	2.030	0.0602	0.030
**Chronic pelvic pain**	1.126	0.0031	0.239
**Dyspareunia**	1.073	0.0011	0.483
**Dysmenorrhea**	0.984	0.0000	0.897
***IV – Impact of age***			
**Symptom**	**Coefficient**	**Adjusted R^2^**	***p*** **-value**
**Dyschezia**	−0.048	0.0298	0.000
**Dysuria**	−0.012	0.0063	0.066
**Chronic pelvic pain**	−0.015	−0.0011	0.448
**Dyspareunia**	−0.062	0.0197	0.004
**Dysmenorrhea**	−0.046	0.0105	0.025

Abbreviations: BMI, body mass index.

**Stage** – The logistic regression showed that the stage of endometriosis could influence the pain associated to dyschezia (OR [odds ratio] = 1.64; *p* < 0.01), and to dysuria (OR = 2.03; *p* = 0.03). The multiple regression, which included age among the independent variables, showed that the ORs were 1.83 for dyschezia, and 2.30 for dysuria.
**Age** – The linear regression showed that age could influence the pain associated to dyschezia (*p* < 0.01; R^2^ = 0.03; β= - 0.04), to dyspareunia (*p* < 0.01; R^2^ = 0.02; β = - 0.06), and to dysmenorrhea (*p* = 0.02; R^2^ = 0.01; β =-0.04).
**Other variables** – The logistic regression showed that smoking habit did not influence the VAS scores (*p *> 0.05 for all symptoms). Similarly, the linear regression showed that BMI did not influence the VAS scores (*p *> 0.05 for all symptoms).

Finally, we have tried to understand if there could be an association between dysmenorrhea (chosen as the reference variable because of its greater pain score) and the other symptoms, using a multiple linear regression model (Tables 4 and 5).

**Table 4:** Results of the multiple linear regression

Symptom	Coefficient (95% CI)	*p*-value
**Dyschezia**	0.12 (−0.02–0.28)	0.09
**Dysuria**	0.05 (−0.24–0.35)	0.71
**Chronic pelvic pain**	0.19 **(0.08–0.29)**	< 0.01
**Dyspareunia**	0.10 **(0.01–0.20)**	0.031

Abbreviations: CI, confidence interval. Adjusted R^2^ 0.06.

**Table 5:** Results of the multiple linear regression (including age among independent variables)

Variable	Coefficient (95% CI)	*p*-value
**Age**	−0.03 (−0.07–0.01)	0.14
**Dyschezia**	0.11 (−0.04–0.27)	0.14
**Dysuria**	0.04 (−0.26–0.34)	0.78
**Chronic Pelvic Pain**	0.19 **(0.08–0.29)**	< 0.01
**Dyspareunia**	0.09 (−0.01–0.18)	0.06

Abbreviations: CI, confidence interval. Adjusted R^2^ 0.06.

Dysmenorrhea is strictly associated with CCP and dyspareunia, but adding age as an independent variable, it was strictly associated only with CPP. Completing the analysis with the Spearman correlation test (Table 6), it was possible to show that dysmenorrhea and CPP are positively correlated (*p* < 0.01; Spearman correlation coefficient [Rho] = 0.20); a positive correlation could also be found between dysmenorrhea and dyspareunia (*p* < 0.01; Rho = 0.15). Moreover, dyschezia and dysuria are positively correlated (*p* < 0.01; Rho = 0.18).

**Table 6:** Correlation matrix among the study variables (dysmenorrhea as reference variable)

	Spearman Rho	*p*-value
**Dyschezia**	0.1248	0.0139
**Dysuria**	0.0894	0.0786
**Chronic Pelvic Pain**	0.2019	0.0001
**Dyspareunia**	0.1506	0.0030

Abbreviations: Rho, Spearman correlation coefficient.

## Discussion

Endometriosis is a complex disease, and pain is an important component of the syndrome, but not the only one.^14^ There is increasing evidence that endometriosis elicits changes in the population of uterine nociceptors. For example, women with endometriosis have many small unmyelinated nerve fibers in the functional layer of their endometrium. These nerve fibers are probably nociceptors, and are not present in women without endometriosis.^15^

Previous studies have shown that there is not an optimal scale to evaluate (and therefore treat) pain related to endometriosis, as pain is a complex and subjective domain. The VAS seems to be the scale with a better balance between strong and weak points.^4^

In the present study, dysmenorrhea was the symptom with the highest VAS score. Previous studies have shown a causal association between severe dysmenorrhea and endometriosis, and this association seems to be independent of the macroscopic type of the lesions or of their anatomical locations, and may be related to recurrent cyclic microbleeding in the implants. Endometriosis-related adhesions may also cause severe dysmenorrhea.^16^ Moreover, the presence of a rectal or vaginal infiltration by posterior deep infiltrating endometriosis (DIE), as well as the extensiveness of adnexal adhesions, are the only factors strictly related to the severity of dysmenorrhea.^17^

As for CPP, it could be considered nowadays a notable health problem that challenges physicians all over the world. This is due to its etiology, which is still unclear, and to the disease itself, which could have a very different presentation (in terms of time onset and severity) among different patients, and the response to treatments is not always successful.^18^ Among the women who underwent laparoscopy for CPP, endometriosis is found in about one third of the cases.^19^

In particular, DIE is the only macroscopic type of endometriosis for which the relationship with CPP symptoms appears to be well understood.^16^ A prompt identification of the lesions, as well as of their localization, is essential for an appropriate decision regarding the management strategy, patient counseling, and preoperative preparation.^20^

In severe pelvic endometriosis, the involvement of the urinary tract is quite common, with subsequent dysuria. Dyschezia, on the other hand, could be considered as a useful predictor for posterior DIE, whose severity is related to the involvement of the posterior vaginal wall, of the rectovaginal septum, and of anterior rectal wall DIE.^17^ ^20^

In the present research, the logistic regression showed the influence of the stage of endometriosis on dyschezia and on dysuria. This is an interesting result because, in previous studies, authors found no association between the type of pain and the stage of the disease.^21^ They stated that the lack of a consistent or strong association between pain and the stage of the disease may be due to the hypothesis that the intensity of the pain is most likely determined by the interaction between endometriotic lesions and sensory afferent nerve fibers, rather than simply by the type and extent of implants alone.^22^ Our results seem to challenge these previous findings.

As for the age variable, other studies showed that endometriosis is more frequent among young women.^23^ Endometriosis, in fact, is a disease that affects almost exclusively women of reproductive age. In fact, its symptoms are strongly linked to the menstrual cycle, a period in which the endometriotic tissue (located outside the endometrium) bleeds, causing swelling and inflammations.

Other studies in the literature demonstrated that the pain caused by endometriosis has been shown to reduce sexual satisfaction and may also lead to a significant impairment in health-related quality of life (QOL) when compared with the general population.^14^ The QOL is very important from the point of view of the patient: for this reason, several studies have been conducted to assess the effects of endometriosis on the physical and not health-related quality of life (HRQoL) of women showing scores similar to those reported in women with cancer (only for the physical domain).^24^ ^25^ ^26^ ^27^ ^28^ The effects were worse in women with severe pelvic pain and advanced disease. Women with DIE have a significant impairment of sexual activity.^29^ We have shown that dysmenorrhea is associated with a greater perception of pain. In these patients, the VAS scale could be used as an evaluation of the follow-up, to assess the impact of the medical therapy on the improvement of the symptoms. In addition, the severity of the stage of endometriosis, correlated with the increase in dyschezia and dysuria, can be used as an indicator for the evaluation of a surgical treatment of endometriosis.

The most important limitations of the present study were: 1) the selection bias of the sample, which could make the sample non-representative and, therefore, render the results ungeneralizable; 2) the type of the study, which did not allow for the temporal and therefore potential causal relation between the variables; 3) the small effect sizes that should be taken in consideration when examining the findings (Annex 1).

As suggested by the scientific literature, it would be interesting to complete the present study by also analyzing the QOL of the patient by using the validated 36-item short form survey (SF-36) questionnaire, which, although it is not disease specific, could be used to add further information, allowing us to measure at the same time both the mental and physical health of the enrolled sample.^29^

**Annex 1:** Definition of symptom

**Constipation**	Constipation is described as a common complication determined by difficult and/or rare passage of stool or both
**Chronic pelvic pain**	Defined as a noncyclical pain lasting for > 6 months that can lead to lower physical performance and quality of life in women
**Dyspareunia**	Dyspareunia is recurrent or persistent pain during sexual activity that causes marked distress or interpersonal conflict
**Dysmenorrhea**	Dysmenorrhea is defined as recurrent common menstrual cramps. Pain usually begins 1 or 2 days before, or when menstrual bleeding starts, and is felt in the lower abdomen, back, or thighs
**Dysuria**	Dysuria is a symptom of pain, discomfort, or burning when urinating

## Conclusion

Concluding, using a validated method, the VAS, we have provided a picture of pain perception of women suffering from endometriosis. We have observed that dysmenorrhea was associated with the highest perception of pain; moreover, it is strictly associated with CCP and dyspareunia. Smoking habit and BMI did not influence the VAS scores. The stage of endometriosis, contrariwise, could influence the pain associated to dyschezia and to dysuria. The age of the patient could influence the pain associated to dyschezia, to dyspareunia, and to dysmenorrhea. One of the most interesting results of the present study was the influence of the stage of endometriosis on dyschezia and on dysuria, which calls into question previous evidence.
